# Editorial for the Special Issue on Micro/Nano-Chip Electrokinetics

**DOI:** 10.3390/mi8050145

**Published:** 2017-05-04

**Authors:** Xiangchun Xuan, Shizhi Qian

**Affiliations:** 1 Department of Mechanical Engineering, Clemson University, Clemson, SC 29634, USA; 2 Department of Mechanical and Aerospace Engineering, Old Dominion University, Norfolk, VA 23529, USA; SQian@odu.edu

Micro/nanofluidics-based lab-on-a-chip devices have found extensive applications in the analysis of chemical and biological samples over the past two decades. Electrokinetics is the method of choice in these micro/nano-chips for transporting, manipulating and sensing various analyte species (e.g., ions, molecules, fluids and particles, etc.) [1,2]. This Special Issue in *Micromachines* is aimed to provide the recent development in the field of Micro/Nano-Chip Electrokinetics. It consists of 15 papers, which cover both fundamentals and applications, original research and reviews. These papers can be classified into four groups as summarized below.

(1) Fundamentals of electrokinetics. Yuan et al. [3] demonstrated a tunable particle focusing in a straight rectangular microchannel with symmetric semicircle obstacle arrays by the use of electrophoretic slip-induced Saffman lift force. Zhou et al. [4] investigated the aggregation of TiO_2_ submicron particles in deionized water under ultra-violet light irradiation and reported a neutralization effect on the particle zeta potential. Bashirzadeh et al. [5] proposed the use of graphite pencil-leads as low cost, disposable electrodes for the study of various electrokinetic phenomena in straight cylindrical microchannels.
(2) Applications of electrokinetics to (bio)particle manipulations. Natu and Martinez-Duarte [6] used numerical simulation to investigate the effects of device geometry and experimental variables on the continuous sorting of neural stem/progenitor cells via streaming dielectrophoresis (DEP). Zhou et al. [7] proposed a microfluidic device with a contraction channel and tested it numerically for the deformability-based particle separation by DC DEP. Zhu et al. [8] demonstrated the use of multiple parallel microchannels in a two-layer stacked microfluidic device for a significantly enhanced throughput in particle and cell manipulation via reservoir-based DEP (rDEP). Li et al. [9] presented a rapid fabrication of high-aspect-ratio 3D hydrogel microstructures using optically induced electrokinetics (OEK).
(3) Applications of electrokinetics to ionic species manipulation. Zhou et al. [10] proposed an electroosmotic flow-based micromixer with an asymmetrical lateral structure for enhanced fluid streams folding and stretching. Mavrogiannis et al. [11] reported a novel microfluidic method for electrokinetic mixing of laminar fluids and controlling of on-chip concentrations using fluidic DEP. Li et al. [12] demonstrated paper-based sample concentration using ion concentration polarization and sample detection with a smart phone. Zhao et al. [13] presented an overview of the various analyte concentration techniques in microfluidic devices with focus on both the physical mechanism and the representative applications.
(4) Other electric field-based applications. Wang et al. [14] investigated the frequency-dependent electroformation of giant unilamellar vesicles in between 3D and 2D microelectrode systems. Liu et al. [15] presented a new method for analyzing the deformability of fused cells under electrical stresses in a microfluidic array device. Tsai et al. [16] studied the effects of system parameters on the power generation by reverse electrodialysis in a microfluidic device with a Nafion ion-selective membrane. Wang et al. [17] developed a microfluidic device for classification of microalgae cells based on the simultaneous detection and analysis of the signals of fluorescence, scattering, and resistance pulse sensing.

We would like to thank all the contributors for submitting their papers to this Special Issue. We also thank all the reviewers for dedicating their time to help improve the quality of the submitted papers.
